# State-Dependent and Bandwidth-Specific Effects of Ketamine and Propofol on Electroencephalographic Complexity in Rats

**DOI:** 10.3389/fnsys.2020.00050

**Published:** 2020-08-11

**Authors:** Michael A. Brito, Duan Li, George A. Mashour, Dinesh Pal

**Affiliations:** ^1^Department of Anesthesiology, University of Michigan, Ann Arbor, MI, United States; ^2^Center for Consciousness Science, University of Michigan, Ann Arbor, MI, United States; ^3^Neuroscience Graduate Program, University of Michigan, Ann Arbor, MI, United States

**Keywords:** anesthesia, electroencephalogram, ketamine, Lempel-Ziv complexity, propofol, rat

## Abstract

There is an ongoing debate as to whether ketamine anesthesia suppresses neurophysiologic complexity at doses sufficient for surgical anesthesia, with previous human studies reporting surrogates of both suppressed and preserved levels of cortical complexity. However, these studies have not assessed cortical dynamics in higher gamma frequencies, which have previously been demonstrated to correlate with the level of consciousness during anesthesia. In this study, we used Lempel-Ziv complexity (LZc) to characterize frontal and parietal electroencephalographic complexity (0.5–175 Hz, 0.5–55 Hz, 65–175 Hz) before, during, and after ketamine or propofol anesthesia in the rat. To control for the potential influence of spectral changes in complexity estimation, LZc was normalized with phase-shuffled surrogate data. We demonstrate that ketamine and propofol anesthesia were characterized by a significant reduction in broadband (0.5–175 Hz) LZc. Further analysis showed that while the reduction of LZc during ketamine anesthesia was significant in 65–175 Hz range, during propofol anesthesia, a significant decrease was observed in 0.5–55 Hz bandwidth. LZc in broadband and 0.5–55 Hz range showed a significant increase during emergence from ketamine anesthesia. Phase-shuffled normalized LZc revealed that (1) decrease in complexity during ketamine and propofol anesthesia—not increase in complexity during emergence—were dissociable from the influence of spectral changes, and (2) reduced LZc during ketamine anesthesia was present across all three bandwidths. Ketamine anesthesia was characterized by reduced complexity in high gamma bandwidth, as reflected in both raw and phase-shuffled normalized LZc, which suggests that reduced high gamma complexity is a neurophysiological feature of ketamine anesthesia.

## Introduction

Although there is still a debate as to the precise neural correlates of consciousness (Boly et al., 2017; Odegaard et al., 2017; Mashour, 2018), several theories are converging upon the idea that the capacity for conscious experience relates to the diversity of states that the brain can generate (Seth et al., 2006; Baars et al., 2013; Tononi et al., 2016; Carhart-Harris, 2018). Analytical measures that capture the complexity of signals such as electroencephalogram (EEG) have been proposed to serve as a surrogate for the diversity of the brain’s repertoire of states and levels of consciousness. Relative to states typically associated with phenomenological content, such as wakefulness or rapid eye movement sleep, complexity is suppressed in association with unconsciousness, such as during slow-wave sleep (Vyazovskiy et al., 2011; Schartner et al., 2017b), coma (Lin et al., 2005; Gosseries et al., 2014), and the anesthetized state (Casali et al., 2013; Sarasso et al., 2015; Schartner et al., 2015; Hudetz and Mashour, 2016; Eagleman et al., 2018, 2019; Pal et al., 2020). Conversely, recent MEG/EEG studies have demonstrated elevated signal diversity induced by canonical serotonergic psychedelics and the dissociative NMDA-antagonist ketamine (Tagliazucchi et al., 2014; Schartner et al., 2017a; Timmermann et al., 2019).

From the perspective of its effects on EEG signal diversity, ketamine diverges from traditional anesthetics at subanesthetic concentrations, as it induces dissociative psychedelic states characterized by a maintained or enhanced repertoire of brain states (Schartner et al., 2017a; Li and Mashour, 2019). This is in contrast to GABAergic anesthetics such as propofol, which have been shown to degrade sensory integration and attenuate neural signal diversity in a dose-dependent manner (Ferenets et al., 2006, 2007; Ishizawa et al., 2016). Furthermore, previous studies have described a remarkably high incidence of subjective reports of dreaming following emergence from ketamine anesthesia that are often described to be vivid, bizarre, and hallucinatory (Sklar et al., 1981; Sarasso et al., 2015). Although dreaming has also been reported in patients receiving GABAergic anesthetic agents such as propofol, the occurrence is reported to be much less common and is often associated with simple phenomenological content more typical of slow-wave sleep (Leslie et al., 2007; Leslie, 2010).

Previous studies have reported both preserved and attenuated measures of spatiotemporal EEG complexity in humans given doses of ketamine titrated to loss of responsiveness (Sarasso et al., 2015; Wang et al., 2017; Li and Mashour, 2019). However, these studies have used human scalp EEG filtered to frequencies of 55 Hz or lower and thus could not characterize the dynamics of higher gamma frequencies. Prior studies from our laboratory have shown suppressed cortical connectivity in the higher gamma (85–155 Hz) bandwidth during sleep and anesthetic (ketamine, propofol, sevoflurane) induced unresponsiveness (Pal et al., 2015, 2016). Thus, cortical dynamics in higher gamma bandwidth may be a more reliable indicator of the level of consciousness. To determine if ketamine anesthesia is associated with a suppression of signal complexity when higher gamma bandwidth is included in the analysis, we utilized intracranial EEG from our previously published studies in rats to assess temporal EEG complexity in three distinct bandwidths (0.5–175 Hz, 0.5–55 Hz, and 65–175 Hz) in frontal and parietal cortices before, during, and after ketamine anesthesia or, as a GABAergic comparator, propofol anesthesia.

## Methods

Adult male Sprague Dawley rats (*n* = 8 for ketamine, *n* = 8 for propofol; Charles River Laboratories) were used for all experiments. The experiments were approved by the Institutional Animal Care and Use Committee and complied with the Guide for the Care and Use of Laboratory Animals. The rats were housed in a temperature-controlled facility with a 12-h light/12-h dark cycle (lights ON at 6:00 AM) with free access to food and drinking water. For detailed methods, see the original studies (Pal et al., 2015, 2016).

### Surgical Procedures

Under surgical isoflurane anesthesia, the rats (*n* = 16) were implanted with stainless steel screw electrodes to record EEG from frontal (Bregma: anterior 3.0 mm, lateral 2.5 mm), parietal (Bregma: posterior 4.0 mm, lateral 2.5 mm), and occipital (Bregma: posterior 8.0 mm, lateral 2.5 mm) cortices. A stainless steel screw electrode over the nasal sinus served as the reference electrode. In a subset of rats (*n* = 8), an indwelling catheter (Micro-Renathane tubing, MRE-040; Braintree Scientific, Braintree, MA, USA) was surgically positioned into the jugular vein to allow for intravenous propofol infusion.

### Electroencephalographic Data Collection Before, During, and After Ketamine or Propofol Anesthesia

**Figure 1:**
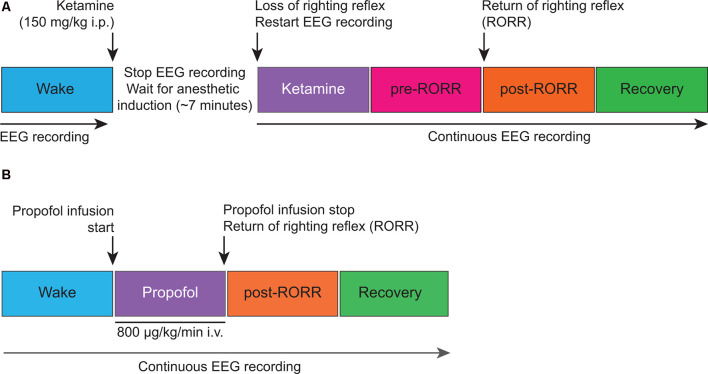
Schematic illustrating the experimental design for electroencephalographic recordings before, during, and after ketamine **(A)** and propofol **(B)** anesthesia. i.p., intraperitoneal; i.v., intravenous.

Monopolar EEG signals—with reference to a screw electrode over nasal sinus—from frontal, parietal, and occipital electrodes were amplified 5,000× (Grass Model 15 LT amplifier system, 15A54 Quad Amplifier; Natus Neurology Inc., Middleton, WI, USA), bandpass filtered between 0.1–300 Hz, and digitized at 1kHz using an MP150 data acquisition unit paired with *AcqKnowledge* software (version 4.1.1; Biopac Systems, Inc., Goleta, CA, USA). The experimental design for ketamine anesthesia experiments is illustrated in Figure 1A. Rats were connected to the recording equipment and baseline wake EEG was recorded for 50 min. After 50 min of baseline data collection, the EEG recording was stopped, and the rats received an intraperitoneal bolus (150 mg/kg) of ketamine hydrochloride. The concentration of ketamine was based on our dose-response experiments, as noted in the original study (Pal et al., 2015), and was also informed by a previously published report (Kelland et al., 1993). After anesthetic induction—as confirmed by the loss of righting reflex (LORR) and the absence of ear, trunk, or whisker movements—EEG data collection was resumed and continued until the completion of the recording session. For EEG analysis, 5-min EEG segments were selected from: (1) last 12.5 min of baseline wake state, (2) first 5 min of ketamine anesthesia, (3) within 12.5 min before the return of righting reflex—RORR—i.e., pre-RORR, and (4) first 12.5 min after RORR, i.e., post-RORR. In three of these rats, EEG was recorded for an extended period and the last 5 min from the recovery period were used for Lempel-Ziv complexity analysis. The experimental design for propofol anesthesia experiments is illustrated in Figure 1B. Baseline wake EEG was recorded for 75 min after which propofol was continuously infused through an intravenous catheter using a microsyringe pump at a rate of 800 μg/kg/min. The concentration of propofol was based on our dose-response experiments, as noted in the original study (Pal et al., 2016). Once LORR was achieved, rats were maintained in a dorsal recumbent position for the remainder of the propofol infusion period. During the anesthesia epochs, rectal temperature was monitored through a rectal probe (RET-2 with Model 7001H, Physitemp Instruments, Clifton, NJ, USA) and the core body temperature was maintained at 37 ± 1^o^C using a near-infrared heating pad (Kent Scientific, Torrington, Connecticut). After 75 min of propofol administration, the anesthetic infusion was stopped and the time of RORR was marked. The EEG recording continued for 2.5 h post-anesthesia. For data analysis, 5-min EEG segments were selected from: (1) last 12.5 min of baseline wake state, (2) last 12.5 min of propofol anesthesia, (3) first 12.5 min period after RORR, i.e., post-RORR, and (4) the last 12.5 min of the recovery phase. Supplementary Figures S1, S2 show the representative EEG traces and spectrograms, respectively, for each state analyzed in ketamine and propofol groups.

### Lempel-Ziv Electroencephalographic Complexity Analysis

We used Lempel-Ziv complexity (LZc)—a surrogate for neural signal diversity—to analyze EEG complexity before, during, and after ketamine and propofol anesthesia (Lempel and Ziv, 1976). LZc analysis was performed as described in previous studies, including from our laboratory (Schartner et al., 2015, 2017a,b; Li and Mashour, 2019; Pal et al., 2020). In brief, the frontal and parietal EEG signals were detrended using local linear regression (10-s window with 5-s overlap). EEG data were lowpass filtered at 175 or 55 Hz *via* a fifth-order Butterworth filter. For analysis of higher gamma frequencies, data were bandpass filtered between 65 Hz and 175 Hz. The filtered signal was then segmented into non-overlapping 10-s windows. The instantaneous amplitude was calculated from the Hilbert transform of the signal, which was then binarized using its mean value as the threshold for each channel. LZc searches for the occurrence of consecutive binary characters, or “words,” and counts the number of times a new “word” is encountered. The resultant LZc values were averaged across all of the windows as an estimate of the temporal complexity at each state. We normalized the original LZc by the mean of the LZc values from *n* = 50 surrogate time series generated by randomly shuffling the binary sequence, as this leads to maximal value for binary sequences for a fixed length. To examine if the complexity as reflected by the LZc is dissociable from the spectral content of the signal, we employed an additional control measure that compared the time-shuffled LZc (as described above) with LZc normalized through phase-randomization (obtained from surrogate data in which the power spectrum of the signal is preserved). The resultant normalized LZc measure (denoted LZc_N_) reflects changes in complexity beyond the spectral content of the signal. Both LZc and LZc_N_ are reported in arbitrary units. It is important to note that Lempel-Ziv complexity is a nonlinear measure that does not require linear assumptions about the structure of the signal (i.e., additivity, stationarity) in the same manner as measures such as spectral power. Therefore, it can be used to derive information from a broader signal band without the need to parse the signal into many individual frequency components, as is traditionally done with spectral power. Our choice of two distinct frequency bands was based on the following considerations: complexity analysis in 0.5–55 Hz frequency band allowed us to have a direct comparison of our rat data to previously reported human data (Sarasso et al., 2015; Wang et al., 2017; Li and Mashour, 2019) and also served as a control to validate our methodology against these previous human studies. The complexity analysis in the higher gamma frequency band was motivated by rat studies from our laboratory (Pal et al., 2015, 2016) that showed high gamma connectivity (above 65 Hz) as a robust indicator of unconsciousness during anesthetic- and sleep-induced unconsciousness.

### Statistical Analysis

All statistical analyses were performed using R software (R Core Team, 2016) in consultation with the Consulting for Statistics, Computing, and Analytics Research Core at the University of Michigan, Ann Arbor. A linear mixed model with fixed effects and an alpha of <0.05 was used to compare LZc values at representative epochs before, during, and after ketamine or propofol anesthesia. Tukey’s *post hoc* test was used to correct for multiple pairwise comparisons across states.

## Results

### Ketamine Shows State-Dependent and Bandwidth-Specific Effects on Lempel-Ziv Complexity

**Figure 2:**
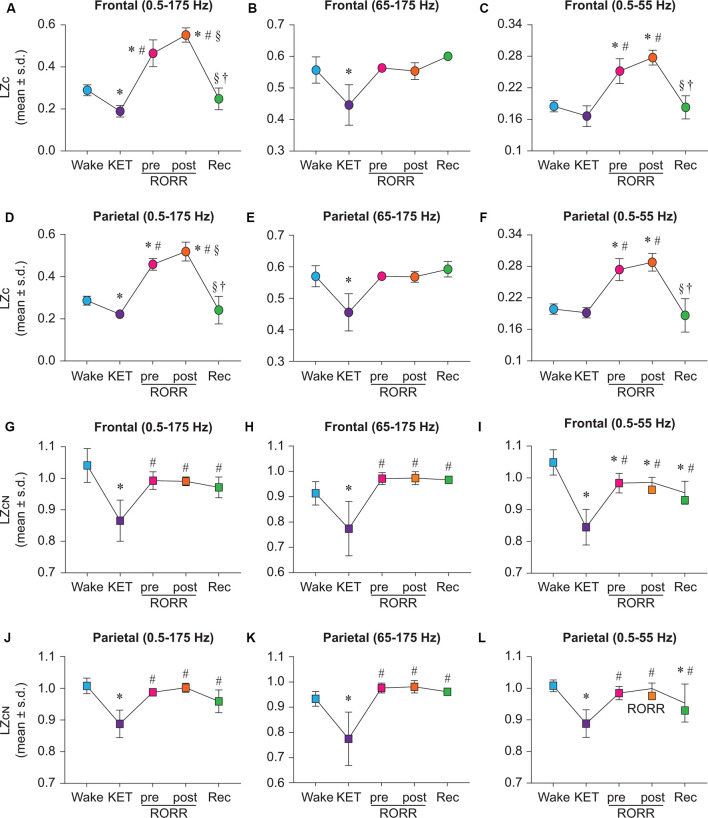
State-dependent and bandwidth-specific effects of ketamine on raw and normalized Lempel-Ziv complexity. Ketamine anesthesia suppressed raw LZc in the broadband range in both the frontal and parietal cortices **(A,D)**. LZc in the 0.5–55 Hz range was not significantly different between wake and ketamine anesthesia in either the frontal or the parietal cortex **(C,F)** while the LZc was significantly decreased in the 65–175 Hz range in both the frontal and parietal **(B,E)** cortices. In contrast, the LZc during emergence from ketamine anesthesia was significantly increased in broadband **(A,D)** and 0.5–55 Hz **(C,F)** range in both frontal and parietal cortices. To assess changes in EEG complexity beyond the spectral content of the signal, we normalized LZc by *n* = 50 phase-shuffled surrogate data. The phase-shuffled complexity (LZc_N_) was significantly decreased across all three frequency bands (0.5–175 Hz, 65–175 Hz, 0.5–55 Hz) in frontal and parietal cortices relative to wake during ketamine anesthesia **(G–L)**. Compared to the waking state, LZc_N_, at emergence did not significantly differ in frontal or parietal cortices in broadband or 65–175 Hz data **(G,H,J,K)**. In 0.5–55 Hz range, LZc_N_ remained significantly attenuated relative to wake during pre-RORR, post-RORR, and recovery wake epochs in the frontal channel **(I)**. In the parietal channel, LZc_N_ was attenuated relative to wake during recovery wake **(L)**. The significance symbols indicate *p*-values at <0.05. The actual *p*-values are reported in the results section. *Significant compared to Wake, ^#^significant compared to ketamine anesthesia (KET), ^§^significant compared to pre-RORR, ^†^significant compared to post-RORR. Tukey’s *post hoc* test was applied to the data to correct for multiple comparisons. s.d., standard deviation; Rec, Recovery wake; RORR, the return of righting reflex.

LZc analysis was first applied to broadband (0.5–175 Hz) EEG data before, during, and after ketamine anesthesia. As compared to baseline wake state, LZc significantly decreased during ketamine anesthesia in both frontal (*t*_(7)_ = −5.432, *p* < 0.001; Figure 2A) and parietal (*t*_(7)_ = −3.998, *p* < 0.001; Figure 2D) areas. During the emergence phase, prior to return of righting reflex (pre-RORR), there was a significant increase in LZc as compared to the baseline wake state (frontal: *t*_(7)_ = 9.432, *p* < 0.001, parietal: *t*_(7)_ = 10.756, *p* < 0.001) and ketamine anesthesia (frontal: *t*_(7)_ = 14.774, *p* < 0.001; parietal: *t*_(7)_ = 14.754, *p* < 0.001; Figures 2A,D). The post-RORR phase was characterized by behavior—hyperactivity, hyperlocomotion, running in circles, ataxia—that is typically reported after administration of subanesthetic concentration of ketamine (Hunt et al., 2006; Hakami et al., 2009). We found the LZc values to be highest during this phase (post-RORR) as compared to wake state (frontal: *t*_(7)_ = 14.097, *p* < 0.001; parietal: *t*_(7)_ = 18.626, *p* < 0.00), and ketamine anesthesia (frontal: *t*_(7)_ = 19.439, *p* < 0.001; parietal: *t*_(7)_ = 18.628, *p* < 0.001; Figures 2A,D). The increase in LZc during post-RORR phase was also significantly higher than the LZc in pre-RORR epoch (frontal: *t*_(7)_ = 4.665, *p* < 0.001; parietal: *t*_(7)_ = 3.872, *p* < 0.01; Figures 2A,D). The recovery wake epoch was characterized by a significant decrease in LZc values as compared to pre-RORR (frontal area: *t*_(2)_ = −8.421, *p* < 0.001; parietal: *t*_(2)_ = −9.934, *p* < 0.001) and post-RORR (frontal: *t*_(2)_ = −11.772, *p* < 0.001; parietal: *t*_(2)_ = −12.745, *p* < 0.001), and returned to the baseline wake state (Figures 2A,D).

To investigate the effect of ketamine on complexity in higher gamma frequencies, LZc analysis was performed on EEG data filtered between 65–175 Hz. As compared to baseline wake state, LZc was significantly reduced during ketamine anesthesia in the 65–175 Hz bandwidth (frontal: *t*_(7)_ = −5.605, *p* < 0.001; parietal: *t*_(7)_ = −7.760, *p* < 0.001; Figures 2B,E). No significant differences in LZc were found between wake, pre-RORR, post-RORR, or recovery wake epochs for frontal (Figure 2B) or parietal (Figure 2E) areas. To compare our data with previously published data from human studies, data were bandpass filtered to 0.5–55 Hz. While the LZc in 0.5–55 Hz band in the ketamine anesthesia epoch was not characterized by any statistical difference as compared to a waking state in frontal and parietal areas (Figures 2C,F), LZc during pre-RORR as well as post-RORR epochs showed a significant increase as compared to a waking state in both frontal (pre-RORR: *t*_(7)_ = 7.993, *p* < 0.001; post-RORR: *t*_(7)_ = 11.042, *p* < 0.001; Figure 2C) and parietal (pre-RORR: *t*_(7)_ = 10.045, *p* < 0.001; post-RORR: *t*_(7)_ = 11.859, *p* < 0.001; Figure 2F), areas. There was no significant difference in LZc between baseline wake and recovery wake epochs (Figures 2C,F).

### Changes in EEG Complexity During Ketamine Anesthesia, but not Emergence, Are Independent of Spectral Changes in the EEG Signal

To determine if the changes in complexity were attributable to the change in the spectral content of the EEG signal, we normalized LZc from all three frequency bands by *n* = 50 surrogate data in which the phases of the signal are randomly shuffled under the constraints of preserving the spectral profile of the original signal. The resultant phase shuffled normalized LZc (denoted LZc_N_), reflects changes in EEG complexity beyond the linear spectral content of the signal.

Similar to the ketamine anesthesia-induced decrease in LZc (Figures 2A,D), LZc_N_ during ketamine anesthesia was also found to be significantly reduced in the broadband frequency range (0.5–175 Hz) in both frontal (*t*_(7)_ = −9.102, *p* < 0.001, compared to baseline wake; Figure 2G) and parietal (*t*_(7)_ = −9.037, *p* < 0.001, compared to baseline wake; Figure 2J) areas. However, the phase-shuffled normalization of LZc attenuated the increase observed earlier for raw complexity during emergence phase (i.e., pre-RORR and post-RORR as in Figures 2A,D), and no significant differences in LZc_N_ were found between baseline wake state, emergence phase (pre-RORR and post-RORR) and the recovery wake epoch (Figures 2G,J). Compared to ketamine anesthesia, there was a significant increase in LZc_N_ during pre-RORR (frontal: *t*_(7)_ = 6.599, *p* < 0.001; parietal: *t*_(7)_ = 7.051, *p* < 0.001), post-RORR (frontal *t*_(7)_ = 6.493, *p* < 0.001; parietal: *t*_(7)_ = 7.854, *p* < 0.001), and recovery wake (frontal: *t*_(2)_ = 4.066, *p* < 0.01; parietal: *t*_(2)_ = 3.703, *p* < 0.01; Figures 2G,J). The LZc_N_ in higher gamma frequency range (65–175 Hz) decreased during ketamine anesthesia (frontal: *t*_(7)_ = −4.748, *p* < 0.001; parietal: *t*_(7)_ = −5.570, *p* < 0.001) compared to wake, while there was no significant difference in LZc_N_ between baseline wake, emergence phase, and the recovery wake states (Figures 2H,K). Compared to ketamine anesthesia, there was a significant increase in LZc_N_ during pre-RORR (frontal: *t*_(7)_ = 6.720, *p* < 0.001; parietal: *t*_(7)_ = 7.421, *p* < 0.001), post-RORR (frontal: *t*_(7)_ = 6.791, *p* < 0.001; parietal: *t*_(7)_ = 7.505, *p* < 0.001), and recovery wake epoch (frontal: *t*_(2)_ = 4.844, *p* < 0.001; parietal: *t*_(2)_ = 4.969, *p* < 0.001; Figures 2H,K). In the bandwidth of 0.5–55 Hz, LZc_N_ was significantly lower than wake during ketamine anesthesia (frontal: *t*_(7)_ = −4.748, *p* < 0.001; parietal: *t*_(7)_ = −4.748, *p* < 0.001; Figures 2I,L). Compared to LZc_N_ during baseline wake state, frontal LZc_N_ remained significantly attenuated throughout the remainder of the experiment (pre-RORR: *t*_(7)_ = −3.972, *p* < 0.01; post-RORR: *t*_(7)_ = −3.835, *p* < 0.01; recovery wake: *t*_(7)_ = −4.055, *p* < 0.01; Figure 2I) while parietal LZc was only significantly lower during recovery wake epoch (*t*_(2)_ = −3.01, *p* < 0.05; Figure 2L). Compared to ketamine anesthesia, there was a significant increase in LZc_N_ during pre-RORR (frontal *t*_(7)_ = 8.46, *p* < 0.001; parietal: *t*_(7)_ = 8.646, *p* < 0.001), post-RORR (frontal *t*_(7)_ = 8.596, *p* < 0.001; parietal: *t*_(7)_ = 9.921, *p* < 0.001), and recovery wake epoch (frontal: *t*_(2)_ = 4.835, *p* < 0.001; parietal: *t*_(2)_ = 4.584, *p* < 0.001; Figures 2I,L).

### Propofol Shows State-Dependent and Bandwidth-Specific Effects on Lempel-Ziv Complexity

**Figure 3:**
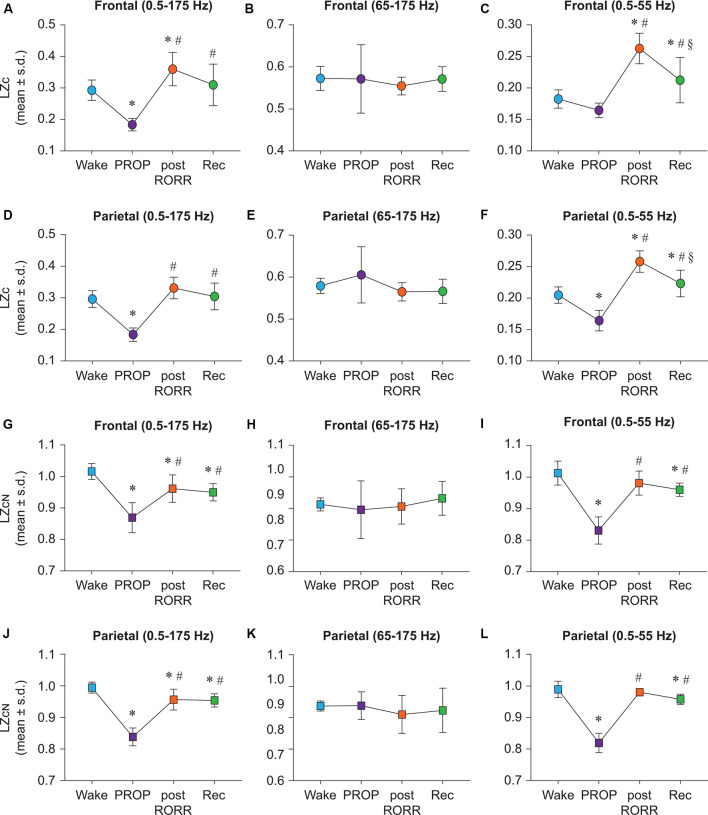
Propofol shows distinct state-dependent and bandwidth-specific effects on raw and normalized Lempel-Ziv complexity. Propofol anesthesia suppressed broadband EEG complexity in both frontal and parietal cortices relative to wake **(A,D)**. Frontal LZc in the 0.5–55 Hz range was not significantly different between wake and propofol anesthesia **(C)** while parietal LZc was significantly reduced **(F)**. Post-RORR was characterized by a significant increase in LZc in frontal broadband EEG complexity **(A)**. In the 0.5–55 Hz band, significant increases in LZc were seen during post-RORR and recovery wake in frontal and parietal cortices **(C,F)**. Propofol administration did not have a significant effect on LZc in the 65–175 Hz range in any of the states **(B,E)**. To assess changes in LZc beyond the spectral content of the signal, we normalized LZc by *n* = 50 phase-shuffled surrogate data (LZc_N_). Propofol anesthesia resulted in significant attenuation in LZc_N_ in frontal and parietal cortices relative to wake in broadband **(G,J)** and 0.5–55 Hz data **(I,L)**. LZc_N_ did not significantly differ between any states in the 65–175 Hz range **(H,K)**. There was no difference in LZc_N_ between wake state and post-RORR in frontal or parietal areas in the 0.5–55 Hz range **(I,L**). The significance symbols indicate *p*-values at <0.05. The actual *p*-values are reported in the results section. *Significantly different from Wake, ^#^significantly from propofol anesthesia (PROP), ^§^significantly different from post-RORR. Tukey’s *post hoc* test was applied to the data to correct for multiple comparisons. s.d., standard deviation; Rec, Recovery wake; RORR, the return of righting reflex.

Propofol anesthesia was marked by a significant reduction in broadband LZc relative to wake state across both frontal (*t*_(7)_ = −5.058, *p* < 0.001; Figure 3A) and parietal (*t*_(7)_ = −7.264, *p* < 0.001) areas (Figure 3D). As compared to baseline wake state, the post-RORR epoch showed a significant increase in LZc values in frontal (*t*_(7)_ = 3.111, *p* < 0.05), but not parietal (*t*_(7)_ = 2.480, *p* = 0.09) areas (Figures 3A,D). Compared to propofol anesthesia, there was a significant increase in LZc during post-RORR (frontal: *t*_(7)_ = 8.169, *p* < 0.001; parietal: *t*_(7)_ = 9.744, *p* < 0.001) and recovery wake epoch (frontal: *t*_(7)_ = 6.692, *p* < 0.001; parietal: *t*_(7)_ = 8.824, *p* < 0.001; Figures 3A,D). There was no significant difference in LZc between baseline wake and recovery wake epoch in both frontal and parietal areas (Figures 3A,D). LZc analysis in the frequency range of 65–175 Hz showed no significant changes in EEG complexity between any of the states in frontal or parietal areas (Figures 3B,E). LZc analysis restricted to 0.5–55 Hz band showed differential effects of propofol on signal diversity that depended on the channel location and state. LZc in the frontal area during propofol anesthesia was not significantly different as compared to LZc during wake state (Figure 3C) while parietal LZc showed a significant decrease (*t*_(7)_ = −6.55, *p* < 0.001; Figure 3F). As compared to wake state, significant increase in frontal LZc was found in post-RORR (*t*_(7)_ = 7.801, *p* < 0.001) and recovery wake epoch (*t*_(7)_ = 2.9, *p* < 0.05; Figure 3C). In the parietal channel, signal diversity was also increased compared to wake during post-RORR (*t*_(7)_ = 9.776, *p* < 0.001) and recovery epoch (*t*_(7)_ = 3.27, *p* < 0.05; Figure 3F). Compared to propofol anesthesia, there was a significant increase in LZc during post-RORR (frontal: *t*_(7)_ = 9.553, *p* < 0.001; parietal: *t*_(7)_ = 16.327, *p* < 0.001) and recovery wake epoch (frontal: *t*_(7)_ = 4.652, *p* < 0.001; parietal: *t*_(7)_ = 9.821, *p* < 0001; Figures 3C,F).

### Changes in EEG Complexity During Propofol Anesthesia, but not Emergence, Are Independent of Spectral Changes in the EEG Signal

In order to determine changes in EEG complexity beyond the spectral content of the signal, we normalized propofol LZc data as previously described for ketamine group. LZc_N_ was found to be suppressed during propofol anesthesia in frontal and parietal channels in the broadband (frontal: *t*_(7)_ = −7.583, *p* < 0.001; parietal: *t*_(7)_ = −12.915, *p* < 0.001; Figures 3G,J) and 0.5–55 Hz (frontal: *t*_(7)_ = −10.096, *p* < 0.001, parietal: *t*_(7)_ = −15.343, *p* < 0.001) bandwidth (Figures 3I,L). As compared to propofol anesthesia, post-RORR was characterized by significantly higher LZc_N_ in broadband (frontal: *t*_(7)_ = 4.937, *p* < 0.001; parietal: *t*_(7)_ = 0.764, *p* < 0.001; Figures 3G,J) and 0.5–55 Hz (frontal: *t*_(7)_ = 8.342, *p* < 0.001, parietal: *t*_(7)_ = 14.531, *p* < 0.001) range (Figures 3I,L). LZcN was also significantly increased from propofol anesthesia during recovery in broadband (frontal: *t*_(7)_ = 4.314, *p* < 0.01, parietal: *t*_(7)_ = 9.561, *p* < 0.001; Figures 3G,J) and 0.5–55 Hz (frontal: *t*_(7)_ = 7.162, *p* < 0.001; parietal: *t*_(7)_ = 12.502, *p* < 0.001) range (Figures 3I,L). In the broadband range (0.5–175 Hz), LZc_N_ remained significantly suppressed relative to wake across frontal and parietal channels during post-RORR (frontal: *t*_(7)_ = −2.915, *p* < 0.05, parietal: *t*_(7)_ = −3.15, *p* < 0.05) and recovery wake epoch (frontal: *t*_(7)_ = −3.539, *p* < 0.01; parietal: *t*_(7)_ = −3.53, *p* < 0.05; Figures 3G,J). As compared to wake state, recovery wake epoch in 0.5–55 Hz range showed suppressed LZc_N_ in both frontal (*t*_(7)_ = −2.934, *p* < 0.05) and parietal (*t*_(7)_ = −2.841, *p* < 0.05) areas (Figures 3I,L). There was no significant difference in EEG complexity in 65–175 Hz between any of the states in frontal or parietal areas (Figures 3H,K).

## Discussion

There has been a debate as to whether ketamine anesthesia reduces cortical complexity like traditional GABAergic anesthetics. Previous human studies have utilized scalp EEG to characterize the effects of ketamine anesthesia on cortical dynamics and reported both preserved and attenuated levels of EEG complexity during ketamine-induced unresponsiveness (Sarasso et al., 2015; Li and Mashour, 2019). However, these studies were based on EEG signals that were low pass filtered at 55 Hz and lacked cortical dynamics in higher gamma frequencies. Using intracranial EEG data from frontal and parietal cortices of rats receiving ketamine or propofol anesthesia, we demonstrate a reduction in broadband (0.5–175 Hz) EEG complexity during ketamine anesthesia that is comparable to that induced by GABAergic anesthetic propofol. Bandwidth-specific analysis restricted to higher gamma frequencies showed that ketamine anesthesia is distinguished from propofol by suppression of EEG complexity in high gamma frequencies in the range of 65–175 Hz, which previous human studies using scalp EEG could not reveal.

To determine if changes in complexity across all states were attributable to processes beyond the spectral content of the signal, we normalized LZc by surrogate data in which the phase information of the EEG was shuffled under the constraints of preserving the original spectral content of the signal. The resultant measure, LZc_N_, has been previously used to reflect changes in EEG complexity beyond the spectral profile of the signal (Schartner et al., 2015, 2017a,b; Timmermann et al., 2019; Pal et al., 2020). Normalized LZc data revealed that a reduction in EEG complexity during ketamine and propofol anesthesia was not attributable to changes in the spectral content of the EEG signal. However, increases in raw LZc observed during emergence from propofol and ketamine anesthesia were attenuated by normalization across all studied bandwidths, suggesting that changes in complexity during these states can be largely attributed to changes in the spectral contents of the EEG signal. The spectral and connectivity changes in these data have been reported in our previous studies (Pal et al., 2015, 2016, 2017), which showed a statistically significant increase in high gamma power and coherence during recovery from ketamine and propofol anesthesia (Pal et al., 2015, 2016, 2017). This further confirms that the changes observed in LZc during emergence are likely due to the spectral bias. Of note, the psychedelic state induced by subanesthetic administration of ketamine in humans is associated with increased LZc that has been shown to extend beyond the spectral contents of the signal (Schartner et al., 2017a; Li and Mashour, 2019). These data suggest that dynamics characteristic of reaching subanesthetic concentrations during recovery from ketamine anesthesia might not be equivalent to the administration of subanesthetic concentrations of ketamine. This lack of equivalence could relate to the differences between delirium and an organized psychedelic state.

A reduction in neural complexity across brain networks has been suggested to be a common feature of states associated with unconsciousness (Tononi and Edelman, 1998; Tononi, 2004; Laureys, 2005; Bodart et al., 2017; Mateos et al., 2018). Prior human studies have used low-pass filtered EEG (45 Hz and below) to assess the effect of ketamine anesthesia on indices of EEG signal complexity using LZc and a closely related measure known as the perturbational complexity index (Sarasso et al., 2015; Wang et al., 2017). These studies concluded that ketamine anesthesia failed to reduce neural complexity, attributing this to either a concomitant increase in the entropy of the EEG signal or the presence of a dream-like state of disconnected consciousness. However, a more recent study in humans (Li and Mashour, 2019) suggests that ketamine-induced state transitions are characterized by dynamic features of normal waking consciousness, general anesthesia, and altered states of consciousness, depending on the dose administered and the temporal course of ketamine administration. Ketamine-induced unresponsiveness was found to be marked by an alternating delta-gamma burst EEG pattern (Akeju et al., 2016), creating signatures of both preserved and suppressed EEG complexity (Li and Mashour, 2019). Our data provide evidence that ketamine anesthesia is marked by stable reductions in Lempel-Ziv complexity that are broadly comparable to GABAergic anesthetics. However, these reductions in complexity during anesthesia are masked when higher gamma frequencies above 55 Hz are filtered out from the EEG signal. Furthermore, the lack of controls to account for the influence of the EEG power spectrum on LZc could potentially explain varying results in prior human studies (Sarasso et al., 2015; Wang et al., 2017).

Our limited channel count restricted us from measurements of single-channel temporal complexity. Although temporal LZc can assess cortical complexity concerning the diversity of temporal patterns within spontaneous EEG signals, it cannot assess complexity concerning features such as neural differentiation and functional integration in the same manner as alternative measurements such as the perturbational complexity index. However, measures of temporal complexity have previously been validated in investigations of cortical dynamics as they relate to the level of consciousness during sleep (Abásolo et al., 2015), anesthesia (Schartner et al., 2015; Pal et al., 2020), and psychedelic states (Schartner et al., 2017a; Timmermann et al., 2019). Furthermore, these measurements are more readily implemented in both human and animal models than measures involving perturbation, as these can be applied to spontaneous neural data and do not require substantial averaging of data over many stimulations. Finally, it is important to note that the routes of administration for our comparisons of ketamine and propofol differed, as ketamine anesthesia was induced by a single intraperitoneal bolus while propofol anesthesia was administered through a sustained intravenous infusion. Future studies using intravenous infusion of ketamine and GABAergic anesthetics such as propofol or etomidate are warranted for a more direct comparison of their anesthetic actions on EEG complexity in rodents.

In summary, our data demonstrate that ketamine anesthesia is characterized by a reduction in broadband (0.5–175 Hz) EEG complexity. As opposed to GABAergic anesthetic propofol, ketamine decreases complexity in higher gamma bandwidth (65–175 Hz). However, decreased complexity in the 0.5–55 Hz range is common to both propofol and ketamine anesthesia. Thus, a reduction in neural complexity may only be a coarse shared phenotype of pharmacologically disparate anesthetics. Future studies will need to focus on the distinct molecular drivers that result in bandwidth-specific reductions of complexity during pharmacologically disparate forms of anesthesia, as well as how these bandwidth-specific reductions in complexity relate to broader mechanisms of anesthetic-induced unconsciousness.

## Data Availability Statement

The raw data supporting the conclusions of this article can be made available on request. Requests to access these datasets should be directed to, Dinesh Pal, dineshp@med.umich.edu.

## Ethics Statement

The animal study was reviewed and approved by Institutional Animal Care and Use Committee, University of Michigan, Ann Arbor, Michigan, United States.

## Author Contributions

MB and DL analyzed the data. MB, DL, GM, and DP interpreted the data and wrote the manuscript.

## Conflict of Interest

The authors declare that the research was conducted in the absence of any commercial or financial relationships that could be construed as a potential conflict of interest.

## Supplementary Material

FIGURE S1 Representative electrophysiological traces show frontal-parietal EEG signals before, during, and after ketamine or propofol anesthesia. The left column shows EEG traces from the pre-ketamine awake state (Wake), ketamine anesthesia (ketamine), epoch before the return of righting reflex (pre-RORR), epoch after the return of righting reflex (post-RORR), and post-ketamine recovery wake state (Recovery). The right column shows the EEG traces from the pre-propofol awake state (Wake), propofol anesthesia (Propofol), epoch with the return of righting reflex (post-RORR), and post-propofol recovery wake state (Recovery). RORR, return of righting reflex.

FIGURE S2 Representative spectrograms show normalized power distribution between 0.5 and 175 Hz during the behavioral states analyzed before, during, and after ketamine **(A)**, and propofol **(B)** anesthesia. Each data epoch separated by black vertical lines is 5 min in length. Both ketamine **(A)** and propofol **(B)** anesthesia epochs show an increase in low frequency (<4 Hz) power and a decrease in high gamma frequencies (65–175 Hz). The emergence from ketamine **(A)** anesthesia (pre-RORR and post-RORR) is characterized by the appearance of an intense increase in high gamma (65–175 Hz) power, that is not seen during either wake or the recovery epoch. The return of righting reflex after propofol anesthesia (post-RORR) show increased activity in theta (4–10 Hz) and gamma power (65–125 Hz).
