# Supplementary figures and images for: State-Dependent and Bandwidth-Specific Effects of Ketamine and Propofol on Electroencephalographic Complexity in Rats
Source: Front Syst Neurosci. 2020 Aug 11;14:50. doi: 10.3389/fnsys.2020.00050 (PMC7431468; doi:10.3389/fnsys.2020.00050)

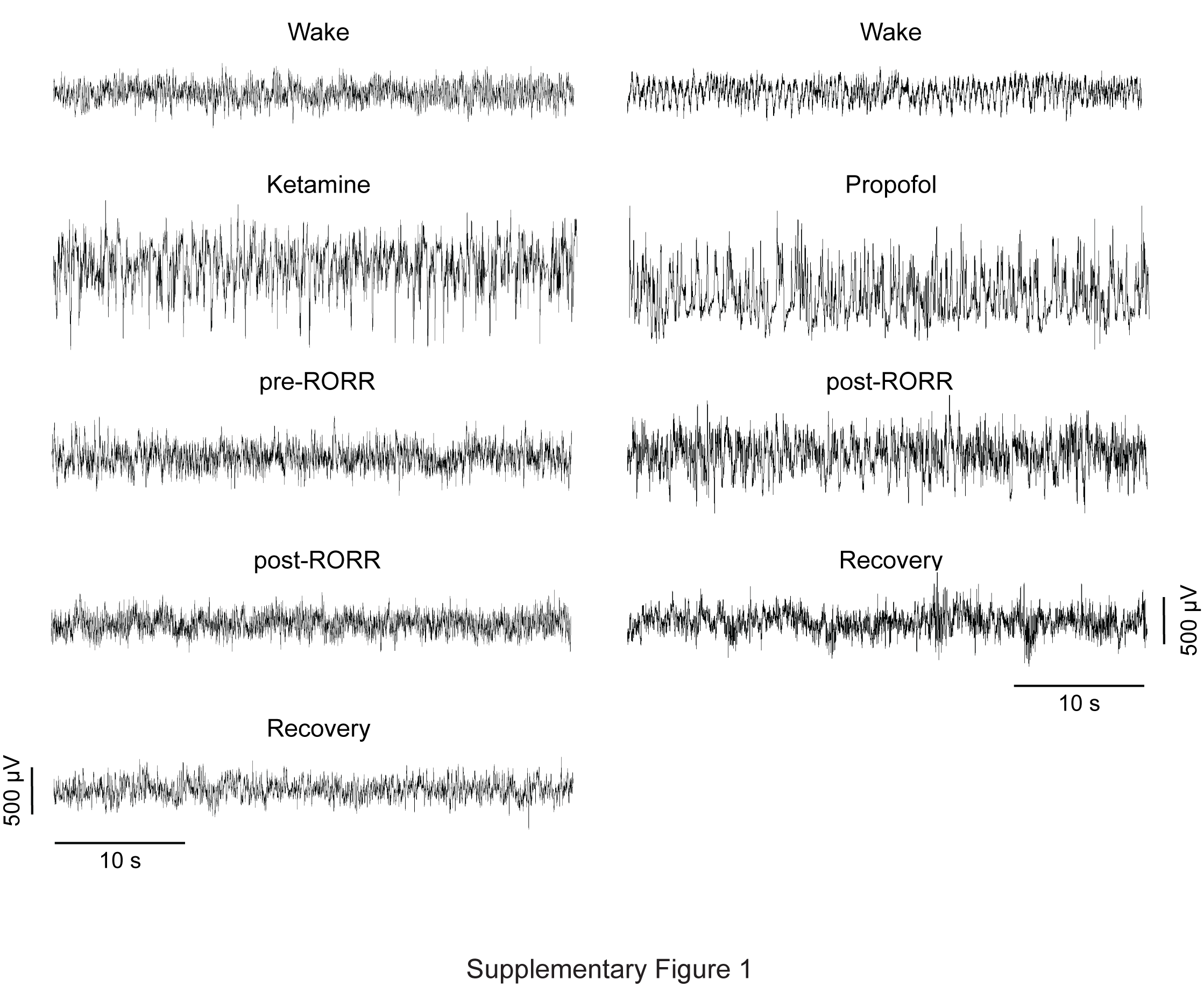

Supplement: FIGURE S1 — Representative electrophysiological traces show frontal-parietal EEG signals before, during, and after ketamine or propofol anesthesia. The left column shows EEG traces from the pre-ketamine awake state (Wake), ketamine anesthesia (ketamine), epoch before the return of righting reflex (pre-RORR), epoch after the return of righting reflex (post-RORR), and post-ketamine recovery wake state (Recovery). The right column shows the EEG traces from the pre-propofol awake state (Wake), propofol anesthesia (Propofol), epoch with the return of righting reflex (post-RORR), and post-propofol recovery wake state (Recovery). RORR, return of righting reflex. [file Image_1.TIF]

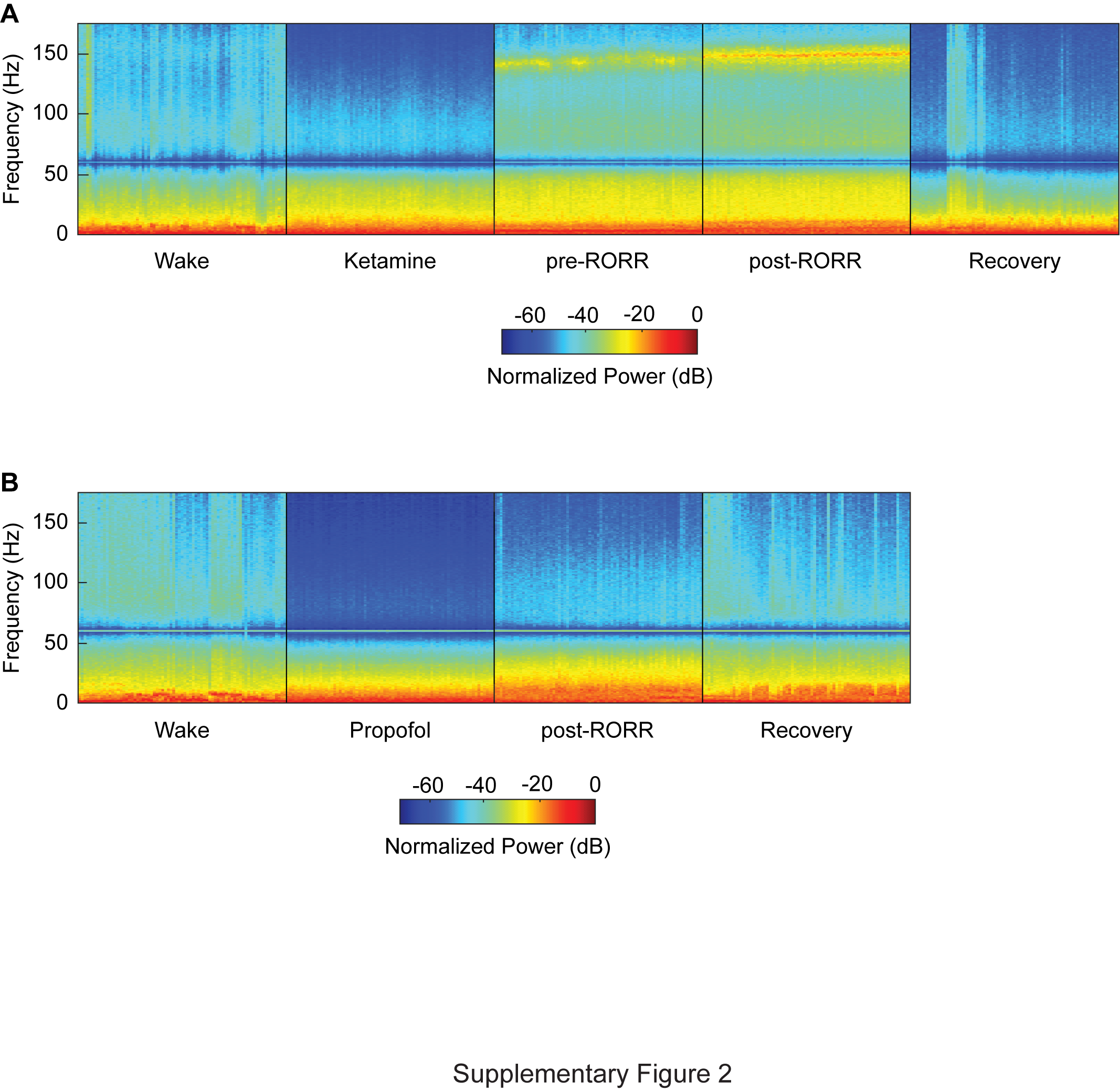

Supplement: FIGURE S2 — Representative spectrograms show normalized power distribution between 0.5 and 175 Hz during the behavioral states analyzed before, during, and after ketamine (A), and propofol (B) anesthesia. Each data epoch separated by black vertical lines is 5 min in length. Both ketamine (A) and propofol (B) anesthesia epochs show an increase in low frequency (<4 Hz) power and a decrease in high gamma frequencies (65–175 Hz). The emergence from ketamine (A) anesthesia (pre-RORR and post-RORR) is characterized by the appearance of an intense increase in high gamma (65–175 Hz) power, that is not seen during either wake or the recovery epoch. The return of righting reflex after propofol anesthesia (post-RORR) show increased activity in theta (4–10 Hz) and gamma power (65–125 Hz). [file Image_2.TIF]
